# The dominant mesopredator and savanna formations shape the distribution of the rare northern tiger cat (*Leopardus tigrinus*) in the Amazon

**DOI:** 10.1038/s41598-022-21412-z

**Published:** 2022-11-04

**Authors:** Tadeu G. de Oliveira, Lester A. Fox-Rosales, Evi A. D. Paemelaere, Katia Maria Paschoaletto Micchi de Barros Ferraz

**Affiliations:** 1 Departamento de Biologia, Universidade Estadual do Maranhão (UEMA), Campus Paulo VI, Av. Lourenço Vieira da Silva 1000, Jardim São Cristóvão, São Luís, Maranhão 65055-310 Brazil; 2 Instituto Pró-Carnívoros, Av. Horácio Neto, 1020, Parque Edmundo Zanoni, Atibaia, São Paulo 12945-010 Brazil; 3 Programa de Pós-Graduação em Ecologia e Conservação da Biodiversidade PPGECB/PPG Em Ciência Animal da Universidade Estadual Do Maranhão, Cidade Universitária Paulo VI, Av. Lourenço Vieira da Silva, nº 1000 – Bairro, Jardim São Cristóvão, São Luís, MA CEP: 65055-310 Brazil; 4 Department of Conservation Biology, Georg-August Universität, Bürgerstrasse 50, 37073 Göttingen, Germany; 5 Panthera, 8 W 40th St. 18th Floor, New York, NY 10018 USA; 6 People and Wildlife Solutions, Manari, Region 9 Guyana; 7 Departamento de Ciências Florestais, Escola Superior de Agricultura Luiz de Queiroz, Universidade de São Paulo, Avenida Pádua Dias 11, Piracicaba, São Paulo 13418-900 Brazil

**Keywords:** Ecology, Zoology, Ecology

## Abstract

Understanding the distribution patterns of threatened species is central to conservation. The Amazonian distribution of the northern tiger cat (N-tiger cat, *Leopardus tigrinus*) and its interspecific relationship with the ocelot, its potential intraguild killer, are intriguing. Here, we combined presence/absence records with species distribution models (SDMs) to determine N-tiger cat occurrence in the Amazon. We also modeled ocelot density from 46 published estimates. The N-tiger cat’s presence in the Amazon was negatively influenced by ocelot density and net primary productivity and positively influenced by savannas and precipitation in the driest month. The best-fitting model predicted highly patchy N-tiger cat occurrence over an area of 236,238.67 km^2^, almost exclusively in savanna enclaves. Additionally, 312,348 camera trap-days at 49 sites in the Amazon revealed no N-tiger cats. The ocelot densities were significantly higher in areas with denser vegetation cover and warmer habitats, with predicted densities ≥ 0.6 ind/km^2^ throughout most of the biome. The lowest ocelot densities (≤ 0.06 ind/km^2^) were observed along the predicted range of N-tiger cats. Our findings highlight that the N-tiger cat’s presence in the Amazon is restricted to savannas and highly influenced by ocelot density, emphasizing the importance of including species interactions in SDMs.

## Introduction

The distribution range of a species has implications for its conservation status. Additionally, it is a crucial attribute of a species’ natural history and is determined by a set of environmental and biotic characteristics. Several developments in species distribution models (SDMs) have resulted in inferring species' ranges from their ecological requirements. These requirements could be influenced by rainfall, vegetation, habitat features, and many other abiotic variables, as well as other species, whether mutualists, competitors or predators^1,2^. Failure to account for species interactions in SDMs has been proposed as a potential cause of poor model performance when predicting species distributions at larger scales^3^.

Predation and competitive displacement by the largest and behaviorally most dominant species affect the distribution, behavior and population sizes of subordinate carnivores^4,5^. Thus, intraguild predation and interspecific killing can drive the structure of carnivore assemblages. In the Neotropical realm, this phenomenon has been demonstrated by ocelot abundance negatively affecting smaller sympatric felid species through direct interspecific killing or behavioral/ecological changes made by the smaller species to avoid ocelots, the so-called ‘ocelot effect’^6^. Thus, modeling this interaction is critical because failure to do so may lead to biased inferences of distributions^3^.

For most carnivore species whose range encompasses mainly the Amazon, such as the jaguar (*Panthera onca*), margay (*Leopardus wiedii*), ocelot (*Leopardus pardalis*) and tayra (*Eira barbara*), among many others, this biome is the part of their range where they remain best protected^7^. Due mostly to the extent of their range in the Amazon, these species are only classified as Near Threatened (NT) or of Least Concern (LC). Red List assessments of such widespread taxa hardly fulfill the criteria for designation as threatened^8^. The criteria consider both the extent of occurrence (i.e., the area contained within the shortest continuous imaginary boundary that can be drawn to encompass all presence records; EOO) and the area of occupancy (i.e., the area within the EOO that is actually occupied by the species; AOO) to determine total population size, one of the key criteria^8^. This only highlights the importance of a proper, current and refined range assessment, especially of threatened taxa.

The northern tiger cat (N-tiger cat, *Leopardus tigrinus*) (Supplementary Information Fig. S1) is a small (2.4 kg) Neotropical felid whose geographic range and limits, as well as its genetic makeup, remain unclear. This cat is part of a species complex and was recently split from *Leopardus guttulus*, the southern tiger cat^9^. The species complex ranges from Central America to southern Brazil, overlapping in part with the more abundant ocelot^10,11^. Its occurrence in the Amazon seems controversial, with multiple variants of the species’ distribution maps. The Amazon comprises distinct ecological regions, which include varied lowland tropical rainforests, deciduous forests, wetlands, and tropical savannas, among others^12^, with multiple centers of endemism^13^. While some consider the species to be absent or mostly absent throughout the region^9,14^, others classify it as present but with a continuous or patchy distribution^15–17^. Regardless, this felid seems so inherently rare in the Amazon that it has been named “the phantom species” of the Amazon^11^. *L. tigrinus* is globally Vulnerable (VU) on the IUCN Red List and Endangered (EN) in Brazil^11,17^.

Herein, we answer two important questions: (1) What drives the species’ distribution in highly diverse Amazonian environments, i.e., which ecological factors determine its occurrence? And (2) Does the ocelot effect play a role in its distribution in the Amazon?

## Results

We obtained 49 records of N-tiger cats for the entire Amazon biome (see Supplementary Information Table S1). Most were from museum records, but an equally important part came from fieldwork-related activities (Supplementary Information Fig. S2). A great part of this fieldwork (51.85%) took place in savannas and adjacent areas. Additionally, 25.93% occurred in rainforests, and an equal percentage in forests along the arc of deforestation in the eastern Amazon (Supplementary Information Fig. S3).

Despite a herculean effort of 312,348 trap-days at 49 study sites throughout the biome, no records were found among the varied Amazonian forest types (Supplementary Information Table S2). Within the Amazon, only in the savanna of Rupununi was the N-tiger cat recorded by camera trapping, with an effort of 1974 trap days.

**Figure 1:**
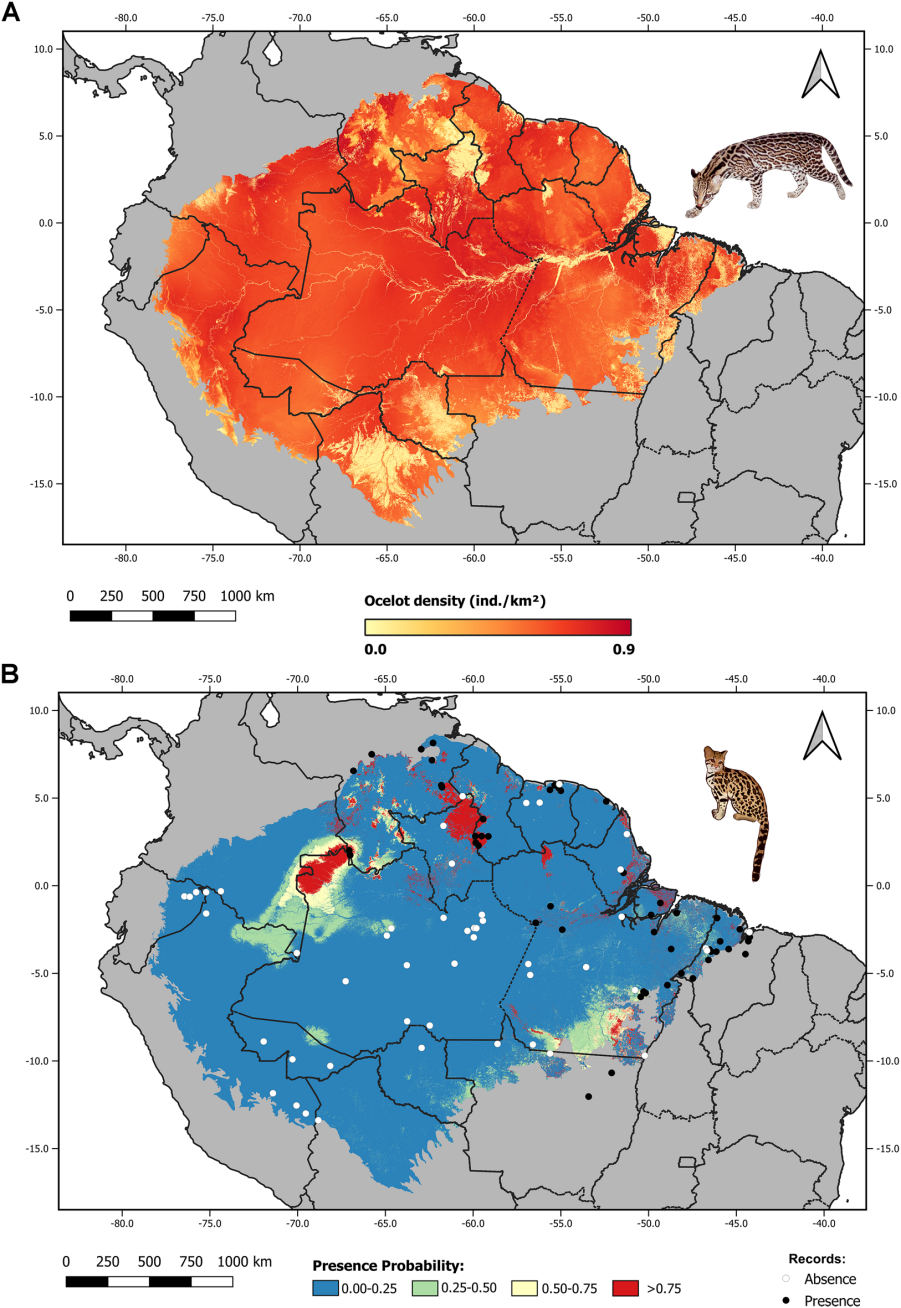
(**A**) Potential ocelot density (ind/km^2^) in the Amazon biome. (**B**) Predicted probability of northern tiger cat presence in the Amazon biome with presence/absence records. Figure made on QGIS ver. 3.4 (www.qgis.org).

Ocelot densities were significantly higher in denser vegetation cover (*P* < 0.001) and warmer habitats (*P* = 0.005). The best model was highly significant (*P* < 0.001) and explained a medium–high level of variability in ocelot density (R^2^ = 0.47, N = 49, Table 1). The model predicted ocelot densities in excess of 0.6 ind/km^2^ throughout most of the biome (Fig. 1A), reaching almost 1 ind/km^2^ in some areas. The lowest ocelot densities were predicted to be along the arc of deforestation, as well as in the Guiana Highlands and savanna ecoregions, reaching densities close to zero in the more open areas (mean = 0.036 ind/km^2^; range = 0.0–0.06 ind/km^2^). Residuals for the best ocelot density model were not correlated (observed = 0.055, expected = − 0.022; *P* = 0.30).

**Table 1:** Parameters of the best-fitting multiple linear regression model assessing ocelot density in the Amazon biome.

Parameter	Estimate	SE ( ±)	t	P
Intercept	− 1.4432	0.1215	− 11.876	< 0.001
TC	0.7590	0.1244	6.104	< 0.001
BIO1	0.3671	0.1244	2.952	0.005

*TC*  percent tree cover, *BIO1* annual mean temperature.

**Figure 2:**
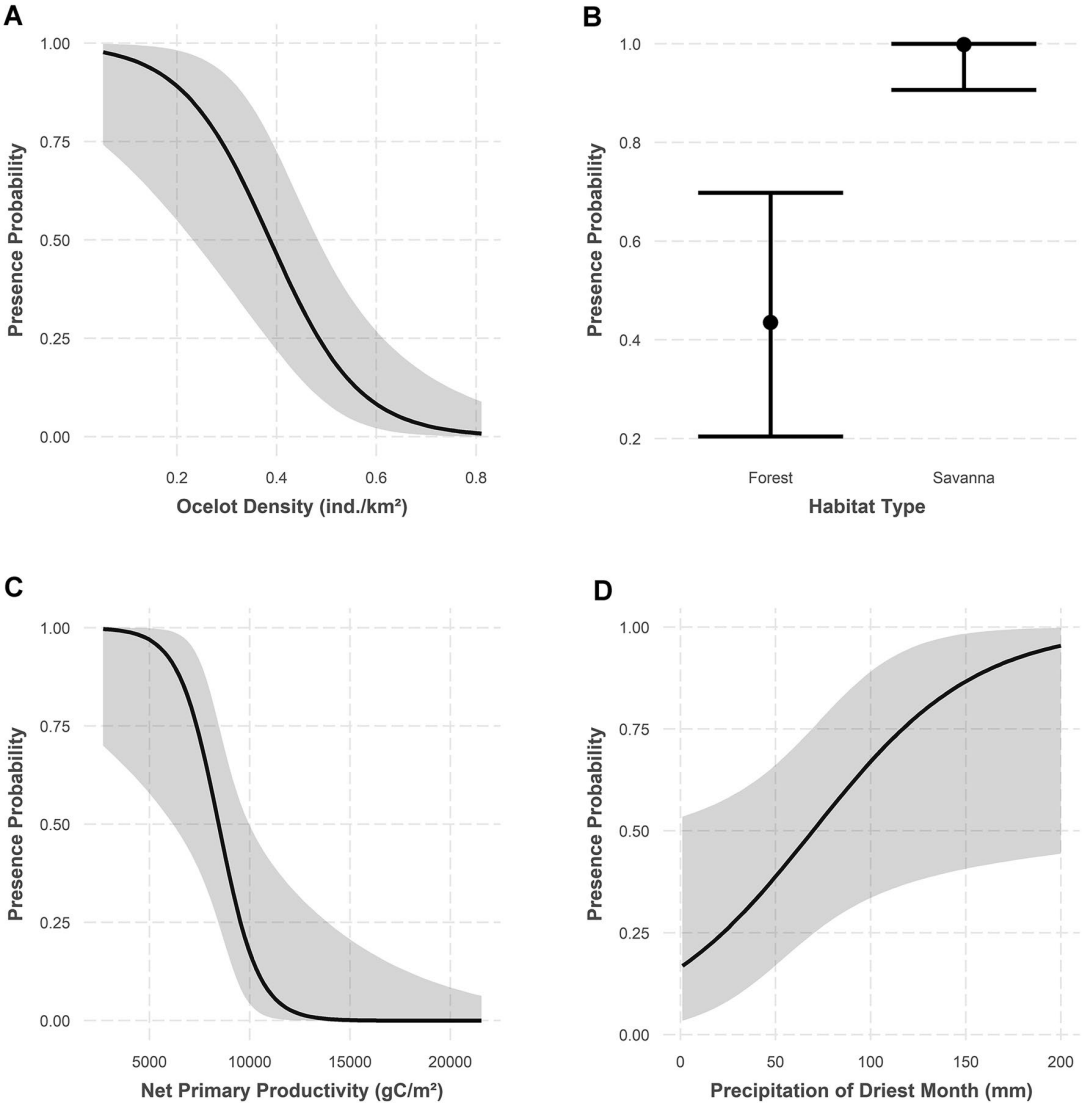
Probability of northern tiger cat presence as a function of different environmental predictors. Gray shading indicates the confidence band at α = 0.05.

**Figure 3:**
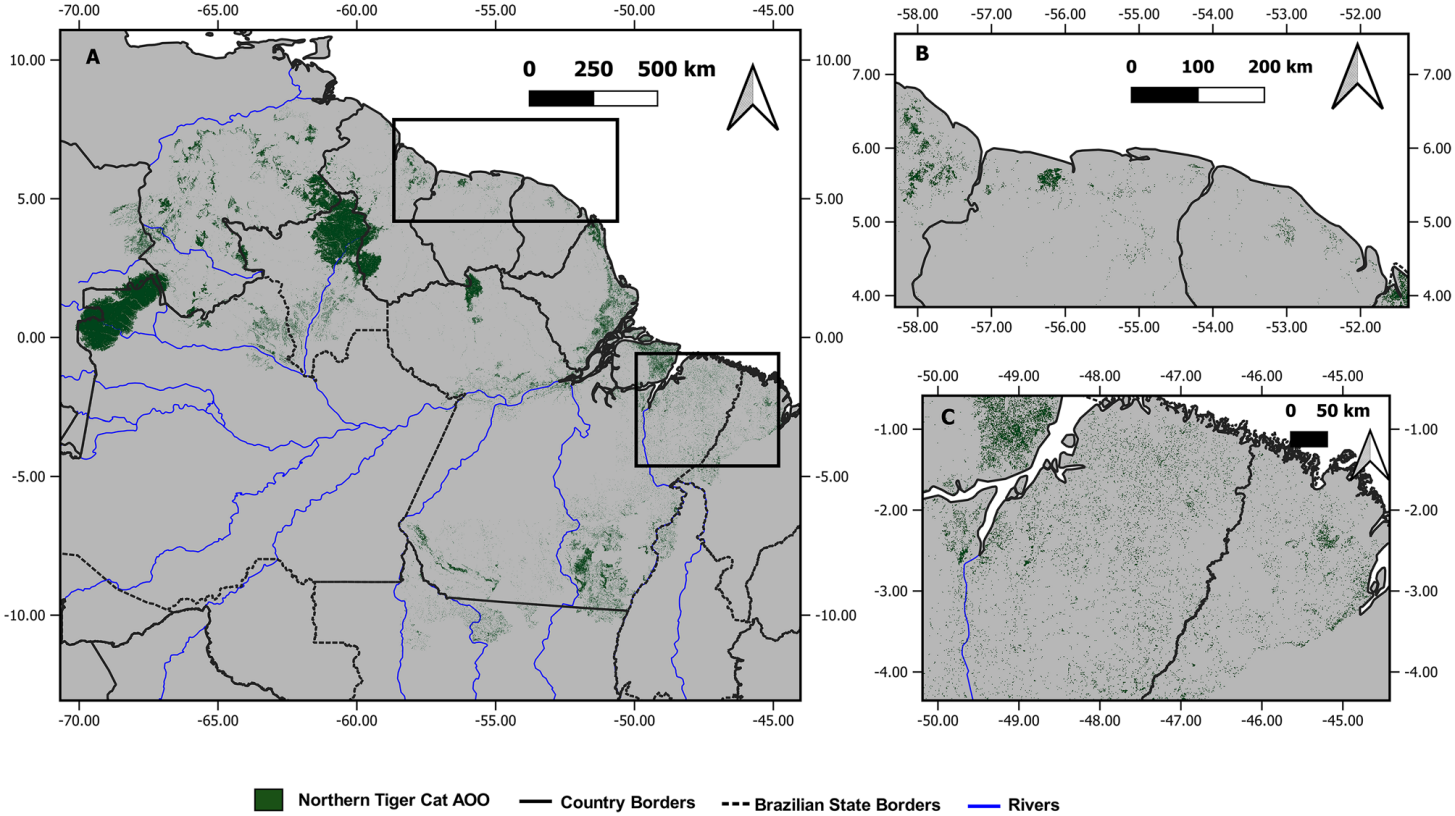
Proposed AOO for the northern tiger cat (*Leopardus tigrinus*) in the Amazon biome using a threshold of 0.75 ind/km^2^. Figure made on QGIS ver. 3.4 (www.qgis.org).

The best-supported N-tiger cat presence model (Table 2) suggests that the species is significantly influenced by ocelot density (*P* < 0.001), savanna formations (*P* = 0.001), net primary productivity (*P* = 0.01), and precipitation in the driest month (*P* = 0.04) and nonsignificantly influenced by the mean diurnal temperature range (Table 3, Fig. 2). The effects of ocelot density and net primary productivity were negative. The best model had a good fit based on a Hosmer–Lemeshow test (*H* = 6.952; *P* = 0.54), with an AUC of 0.95 (Supplementary Information Fig. S4), a sensitivity of 0.83, specificity of 0.92, and a Nagelkerke’s R^2^ of 0.87. The model’s projection for the entire biome suggested high probabilities of N-tiger cat presence in the Guiana Shield and essentially zero probability of its presence throughout most of the Amazon (Fig. 1B). The EOO was estimated to be 383,480.59 km^2^, whereas the AOO was 236,238.67 km^2^ (Fig. 3). Residuals for the best tiger cat presence-absence model were not correlated (observed = − 0.017; expected = − 0.010; *P* = 0.60).

**Table 2:** Model comparisons of the best logistic regression models (ΔAIC ≤ 2.0) for assessing N-tiger cat occurrence in the Amazon biome.

Model	K	AIC	ΔAIC	AIC_W_	LL
LPDENS + SAV + BIO2 + BIO14 + NPP	6	43.5	0	0.28	− 15.74
LPDENS + SAV + BIO14 + NPP	5	44.3	0.79	0.19	− 17.134
LPDENS + SAV + BIO14 + BIO2 + H1 + NPP	7	44.4	0.94	0.17	− 15.207
LPDENS + SAV + NPP	4	44.9	1.36	0.14	− 18.436
LPDENS + SAV + BIO1 + BIO14 + BIO2 + NPP	7	45.1	1.66	0.12	− 15.57
LPDENS + SAV + BIO14 + BIO2 + BIO3 + NPP	7	45.4	1.97	0.1	− 15.723

*AIC = Akaike Information Criterion.

*∆AIC = Change in AIC units with respect to the most parsimonious model.

*AIC_W_ = AIC weight.

*LL = Log-likelihood.

LPDENS = log-transformed ocelot density; SAV = savanna; BIO2 = mean diurnal range; BIO14 = precipitation of driest month; NPP = net primary production; H1 = canopy height; BIO3 = isothermality.

**Table 3:** Parameters of the best-fitting logistic regression model to assess northern tiger cat occurrence in the Amazon biome.

Parameter	Estimate	SE ( ±)	Z	P	Odds ratio
Intercept	− 0.2612	0.5539	− 0.47	0.637233	−
LPDENS	− 3.3416	0.9260	− 3.609	< 0.001	0.0354
SAV	6.6121	2.0778	3.182	0.001	744.04
BIO2	0.82	0.5071	1.616	0.11	2.27
BIO14	1.1488	0.5512	2.084	0.04	3.15
NPP	− 3.66	1.48	− 2.47	0.013	0.026

*LPDENS*  log-transformed ocelot density, *SAV  *savanna, *BIO2*  mean diurnal range, *BIO14*  precipitation of driest month; *NPP*  net primary production.

The average ocelot density in the N-tiger cat EOO was 0.32 ind/km^2^, whereas in its AOO, the average ocelot density was approximately 0.26 ind/km^2^, ranging from 0.63 ind/km^2^ in forests to 0.09 ind/km^2^ in savannas. The percentage of the N-tiger cat AOO within savannas was 74.67%.

## Discussion

Most records of N-tiger cats were from savanna environments, and it was not surprising that this vegetative formation has a key influence on the N-tiger cat range in the Amazon. The bulk of the *L. t. tigrinus* distribution lies in the savannas, dry forests and shrublands of the Cerrado and Caatinga biomes. These are also the areas with the vast majority of records for this lowland subspecies (Supplemental Fig. S5). Hence, *L. t. tigrinus* is more associated with savannas and savanna-like environments than with rainforests. In fact, more than 80% of the records in the Amazon were within 100 km of a savanna patch. Colonization of the northern savanna formations of the Amazon by the N-tiger cat likely occurred during the forest-savanna shifts of the glacial period^18^, and the cat currently shows a patchy distribution. Strong evidence of established biogeographic corridor connections between the savannas of the Cerrado and those of the Amazon exists, suggesting northward expansion of the former during glacial periods, perhaps predating the Last Glacial Maximum^19–21^. Further corroborating this evidence, tiger cat ‘gene flow’ niche modelling showed prior connectivity between the Guiana population and that of Central Brazil and no connectivity with the Andean population^22^. Additionally, Guianan tiger cat skin patterns are found in savanna and transitional savanna/Amazon areas and in the semiarid shrub-woodland of Brazil and are very distinct from the patterns of the tiger cats from the Andes of northwestern South America and Central America (Supplementary Information Fig. S6).

The bioclimatic variables in the best model also supported the cat’s preference for savanna areas. The best model indicated a positive effect of precipitation in the driest month on the probability of the presence of the N-tiger cat, likely indicating the Aw/As climates of tropical savannas^23^. These climates are marked by seasonal variation in rainfall, with a pronounced dry season. Higher rainfall during the dry season favors the growth of vegetation, which results in some tree cover within the savannas. Thus, our results agree with previous research suggesting that tiger cats avoid open savanna formations^24^. Similarly, the species had a significant negative response to net primary productivity. This also supports the species’ avoidance of dense lowland rainforests, which are the most productive habitats. In the Amazon biome, the least productive areas are found in more open landscapes^25^.

The N-tiger cat’s range considered from an ecoregion perspective^12^ could biogeographically explain its distribution in the Amazon. All records but 2 fell within Guiana savannas, Guiana highland forest, Guiana rainforest, part of the Uatumã-Trombetas rainforest bordering the Guianas or all of it connecting to Gurupá and Monte Alegre varzea forests, as well as Marajó varzeas, the interfluve Tocantins-Araguaia/Maranhão, and the southern block of the interfluve Xingu/Tocantins-Araguaia. There were two records from the Negro-Branco moist forest, which also includes savanna-like “campinarana” formations. The range also reaches the transitional babaçu palm forests of Maranhão and the Mato Grosso seasonal forests (Supplementary Information Fig. S7, Table S3). The N-tiger cat’s range in the Amazon was determined by combining records with species distribution modeling, also matching the ecoregion perspective.

Outside the Guiana Shield and likely the savanna patches of the region of the Upper Negro River, in other parts of the Amazon, the N-tiger cat seems to be restricted to the forests of the eastern Amazon, along the arc of deforestation and to transitional areas with savanna formations. The presence and absence points at camera-trapping sites could explain the N-tiger cat’s range in the Amazon and define its distribution range in the biome. Absence points, for instance, were usually located in dense rainforest habitats throughout the Amazon biome.

The species may occasionally occupy rainforests, such as those of the Guianas, where it tends to be very rare. At a site in central Suriname, after an enormous trapping effort of > 20,000 trap days in four years by cat specialists, over an area > 1100 km^2^, no records of the N-tiger cat were found (Supplementary Information Table S2), although its presence is expected in that area^26^. This finding attests to the inherent rarity of this felid in its limited range within the Amazon. However, could its association with the arc of deforestation be related to the replacement of forest by bushy savanna-like vegetation that succeeds abandoned pastures? The other currently recognized subspecies, *L. t. pardinoides* (the Andean tiger cat) and *L. t. oncilla* (the oncilla), and the recently split southern tiger cat *L. guttulus* are all associated with forested areas. Conversely, *L. t. tigrinus* has higher abundance and is mostly found in the nonforested habitats of the Cerrado and Caatinga domains of Brazil and only rarely in rainforests. Thus, *L. t. tigrinus* may be an open-habitat (sub)species. However, within savannas, N-tiger cats are restricted to denser savanna formations, with open savannas deemed unsuitable^24^. In the semiarid Caatinga, the N-tiger cat also prefers denser formations^27,28^.

One of the most interesting findings was the clear relationship between the ranges of the dominant mesopredator and subordinate species. The ranges of ocelots and N-tiger cats in the Amazon were diametrically opposite (Fig. 1), a finding never recorded for felids. The reported ocelot densities and relative abundance indexes (RAIs) in the Amazon range from 0.29 to 0.95 ind/km^2^ and 0.07–13.2 ind/100 trap-days, respectively^7,29^. Thus, the expected ocelot density found using modeling that allows for N-tiger cat presence is very low (Fig. 2A). In the Rupununi, the ocelot:N-tiger cat RAI ratio was roughly 10:1, with a very low RAI and expected density for N-tiger cats (see Supplementary Material). The only other relative abundance estimate of tiger cats presented for the Amazon^30^ was not confirmed as an estimate of tiger cats following inspection of the original records by the authors but as an estimate of margays or ocelots. This antagonistic relationship between ocelots and all other small cat species in their area of sympatry is quite impressive. It is density-dependent, as it seems to take effect only above an ocelot density threshold of 0.12 ind./km^2^^31^. The influence can range from patterns of density, distribution, and occupancy to spatial and temporal use. Conversely, such an impact was not detected when either the small cats or ocelots were compared to the larger cats^31–35^.

In view of the Red List assessments and applying the limited estimates presented, the expected total population size for N-tiger cats in the Amazon would be approximately 150 and 1622 individuals, considering their AOO or EOO, respectively. Applying the IUCN’s formula for mature individuals^8^, these numbers would be 45 and 487 individuals for the AOO and EOO, respectively.

The ocelot’s preference for very dense rainforests may explain the low probability of N-tiger cat occurrence within the Amazon biome. Notably, most tiger cat records from rainforests and all those from premontane forests came from the Guiana Shield, a region where tropical grasslands and savannas dot more forested landscapes. The Guiana Highlands and Pantepui ecoregions, which make up a considerable portion of the shield, tend to have low ocelot densities (below 0.30 ind/km^2^), although they do contain some rainforest. Ocelot densities reach some of their lowest values in the Guianan savanna ecoregion (mean ocelot density of 0.029 in the savanna formations), where the N-tiger cat probability of occurrence was highest. At the Karanambu site in the Rupununi, all ocelot records came from either gallery forests or forest patches embedded in the savanna. Although the data did not allow us to test further hypotheses, it is likely that spatial partitioning occurs in the Guiana Shield, with N-tiger cats favoring habitats that are more open. Conversely, areas farther west in the Amazon biome, other than the predicted area, do not have any major savanna patches and are covered mostly by lowland tropical rainforest formations, where ocelots can potentially reach densities in excess of 0.7 ind/km^2^. Of all Amazonian records of N-tiger cats, only one came from west of the 68th meridian: a preserved specimen from Puerto Leguizamo on the Putumayo River in Colombia. The specimen was identified as *L. t. pardinoides* by its collector, so it most likely represents an individual that came down from the foothills of the Andes. Alternatively, it could have been caught in the Andean foothills but labeled generally as from Puerto Leguizamo, as museum records do not always present precise locations, like most of those from our dataset; thus, they could represent a broader region, not a single collection location.

The records of *L. t. tigrinus* in the Monte-Alegre Várzea ecoregion and Tapajós-Xingu Moist Forest ecoregion (which shares a border with the Amazon River) are actually from the small savanna patches of Terra Santa and Alter do Chão, respectively, which are imbedded within the forests of these ecoregions. Similarly, the Negro-Branco Moist Forest ecoregion includes open-canopy white sand forests with savanna-like vegetation, known as ‘campinaranas’^36^.

Although our model predicted a high probability of N-tiger cat presence in the Marajó Várzea ecoregion, the records from the island came from savanna patches and not from flooded forests and mangroves. Hence, we did not include such large areas in the AOO for the subspecies. It is likely that the highly predicted probability of presence there is an artifact of low predicted ocelot density. Nevertheless, the environment there is not suitable for either cat. Our ocelot density model was highly significant and explained almost 50% of the variation in ocelot density. The remaining variation was related to either other variables that could not be measured via satellite imagery (such as prey availability) or the sampling design of the different studies. Nonetheless, ocelot densities predicted from our model across the Amazon were within the expected range for the species^29^.

Why are N-tiger cats absent in camera-trapping studies in Amazonian forests throughout the biome? The most straightforward answer seems to be because they simply are not there (central and western Amazon) or, where present, their numbers are extremely low (Guianas and eastern Amazon). The lack of surveys cannot be cited as a potential reason for their apparent absence because the studies that did not detect the species were conducted throughout the Amazon biome, in all nine Amazonian countries. Some of the areas have been surveyed for several years—or decades in some cases—and have failed to record a single individual (Supplementary Information Table S2). Typically, N-tiger cats appear, even prominently, on cameras in other biomes, such as in the savannas of the Cerrado and semiarid scrub of the Caatinga domain in Brazil, including sites where ocelots are present^24,27,37^. Clouded tiger cats (*L. t. pardinoides*) have also been frequently recorded on cameras in the Andes, higher than 1500 m above sea level^34,38^, but not in lowland Amazonian forests. This finding indicates that the N-tiger cat is not camera-shy. In northern Brazilian savannas, its density can reach 0.25 ind/km^2^ ^24^. Coincidentally, this highest density estimate of the N-tiger cat is the same as the lowest ocelot density estimate for Amazonian forests^24,29^.

Tiger cats and margays show high similarity, making misidentifications relatively common^39^. However, the evaluation of > 3000 camera trap images of small-medium felids in the Amazon revealed that only one mildly resembled a tiger cat, a finding that supports the species being absent there and does not represent a case of mistaken identity with margays or even ocelots^7^.

The Amazonian range of *L. tigrinus* is very limited, and populations are expected to be very small. With the upcoming split of *L. t. tigrinus* and *L. t. pardinoides* into two different species^40^, this situation would have serious implications for the conservation of the former. Thus, *L. t. tigrinus* conservation lies outside the “Amazonian safe haven” of most other carnivore species found there^7^. The Brazilian drylands Cerrado and Caatinga represent such places for *L. t. tigrinus* populations. Unfortunately, these biomes have had > 50% of their cover completely removed^41^. Very importantly, besides being extremely rare in the Amazonian savannas, this rather limited vegetative formation is also considered highly threatened and of conservation priority^42^. Therefore, the tiger cat could become an emblematic flagship species representing the uniqueness of this vegetative formation in dire need of protection.

In short, the picture that emerges is that although the N-tiger cat uses both rainforests and deciduous forests in the Amazon, it seems to be mostly associated with savanna formations and that its distribution in the Amazon is highly influenced by the ocelot, the dominant mesopredator. The N-tiger cat’s inherent rarity, expected population size, and restricted range in the Amazon suggest that this biome does not in fact represent a safe haven for the global conservation of this small felid. In addition to shedding light on and refining the N-tiger cat distribution in the Amazon, this paper highlights the importance of including biological variables, such as the potential impacts of competitors and predators on species presence and distribution, in SDMs.

## Materials and methods

### Northern tiger cat presence/absence points

We gathered all confirmed records of tiger cat presence that we could find in the Amazon. This scarce material came from specimens deposited in museum collections worldwide, from field data from a few visual identifications, and from incidental traffic-apprehended and road-killed animals in the arc of deforestation in the eastern Amazon. We did not trap live animals in this study. Camera-trapping material came from a study site in the Rupununi savannas of southern Guiana and is described in the supplemental material. As absence points, we classified the data from camera trap studies conducted in the Amazon, with a minimum of ≥ 1900 trap nights that did not record the species (Supplementary Information Table S2). Although we acknowledge that imperfect detection does not necessarily indicate complete absence, the cumulative effort of these studies is substantial, with > 300,000 trap nights covering the entire biome. N-tiger cats readily appear on camera traps if they are present in an area^24,27,37^. With high trapping efforts that can detect rare species (≥ 1900 trap-nights), their lack of detection is practically equal to true absence^43,44^.

We used the exact coordinates of the presence records. We georeferenced museum records and road kills to the nearest patch of natural vegetation of the given location, assuming that no significant environmental variation exists between the location and the georeferenced record. Most museum records provided reference points and distances, meaning our georeferenced locations are not likely to be far from the actual collection site (error margin in the tens of km rather than in the hundreds). As all points were relatively distant (distance between nearest points ~ 19 km), we assumed no spatial correlation and did not apply spatial filters. However, we did test for spatial correlation of residuals after running the models (see below). As such, all available records were used in the modeling. Although some of the museum specimens dated back to the last century, they all came from areas that have not undergone extensive habitat loss (i.e., savannas in the Rupununi and on Marajó Island); hence, we did not apply temporal filters to the data and assumed that the species is still present at all these locations. Records from the deforestation arc (where habitat loss is ongoing at a rapid rate) were all from field observations, roadkill, and animal apprehensions.

### Modeling ocelot density

We modeled the ocelot density to assess its possible effects on N-tiger cat occurrence in the Amazon, using 46 density estimates that were available for the species (Supplementary Information Table S3). The multiple linear regression model incorporated 23 environmental variables (Supplementary Information Table S4, for a detailed description and references), of which 19 were bioclimatic variables from the WorldClim database, version 2^45^. The other four variables were as follows: (i) mean tree cover, which is positively correlated with ocelot presence^30,46^; (ii) mean elevation above sea level, which is negatively correlated with ocelot presence^47^; (iii) mean net primary productivity^25^, the rate at which energy is stored as biomass by producers and a potential proxy for herbivore abundance^48^; and (iv) mean canopy height^49^, which at high values is representative of dense forest cover, the vegetation type preferred by ocelots^50^.

We standardized all predictors to a spatial resolution of 1 km^2^. Correlations between environmental variables were evaluated through Pearson coefficients using a cutoff value of r ≥ 0.70, discarding the variable that was less correlated with the dependent variable (ocelot density). All predictors were converted into z scores, and ocelot densities were log-transformed. Models were ranked according to the Akaike Information Criterion (AIC). Regression assumptions of the best density model were evaluated by examining residual plots (residuals versus fitted values), histograms, and QQ plots. The best density model was projected for the entire Amazon biome. Spatial autocorrelation of model residuals was assessed with a Moran’s I test, using packages spdep^51^ and ape^52^. This test estimates the coefficient of spatial overlap between data points by comparing the observed coefficient against an expected coefficient under a null hypothesis of no spatial correlation. A significant difference between both coefficients indicates that there is evidence of spatial correlation in the model residuals. All analyses were run in R software ver. 3.3.6^53^.

### Modeling northern tiger cat presence

We modeled N-tiger cat presence using generalized linear models (error family = binomial). This framework of modeling uses presence-absence data of the species of interest as a function of predictor variables.

The same 23 environmental variables from the ocelot density model were included, plus two additional variables: savanna vegetation and ocelot density. Since approximately half of the records came from savanna habitats, we considered savanna presence a possible predictor of tiger cat presence in the biome. Similarly, we also modeled the negative intraguild interaction of ocelots with tiger cats^6^ using the density map produced in the prior step.

As with the ocelot density model, the weakest correlated variable was removed in the case of highly correlated variable pairs (r ≥ 0.70). A global model including all noncorrelated predictors was run first, and from this model, we constructed further models, retaining predictors whose 85% confidence intervals did not overlap zero^54^. Models were ranked according to the WIC^55^, model fit was assessed using a Hosmer–Lemeshow test, the area under the receiver operating characteristic curve (AUC) was calculated, and Nagelkerke’s R^2^ was calculated. The model was validated by performing a tenfold cross-validation with 75% of the data for training and 25% for testing, and the AUC was calculated for each validation run. As with the ocelot density model, spatial autocorrelation of model residuals was assessed using the Moran’s I test.

The best model was projected for the entire Amazon biome, and we converted the logit values from the model into probabilities using the equation

$$ P_{\left( x \right)} = \frac{{e^{g\left( x \right)} }}{{1 + e^{g\left( x \right)} }} $$

where ${P}_{\left(x\right)}$ represents the probability of a 1 km^2^ cell being occupied by N-tiger cats. All spatial analyses were conducted with QGIS ver. 3.4^56^. All statistical analyses were conducted using R software ver. 3.3.6^53^ and the packages performance^57^, rcompanion^58^, and ROCR^59^.

### Range delineation

The EOO was estimated using a combination of modeling results and records with environmental features such as rivers and ecoregions^12^. First, we reclassified the spatial projection from our best model using a 50% threshold for the probability of presence^60^. Next, we refined the EOO limit by considering ecoregions, rivers and vegetation formations. This strategy is critical because SDMs often fail to include natural barriers^61^. For the AOO, we considered an expected presence threshold of a higher suitability area (HSA) of 75% as a proxy for the area occupied by the species. Similar to the EOO, possible environmental barriers were considered.

### Ethics approval

No live animals were trapped in this study.

## Supplementary Information

Supplementary Information.

## Data Availability

The datasets used and/or analyzed during the current study are available from the corresponding author upon reasonable request.
