# Supplementary material for: The dominant mesopredator and savanna formations shape the distribution of the rare northern tiger cat (Leopardus tigrinus) in the Amazon
Source: Sci Rep. 2022 Nov 4;12:18697. doi: 10.1038/s41598-022-21412-z (PMC9636236; doi:10.1038/s41598-022-21412-z)
Supplement: Supplementary file 1 — Supplementary Information. [file 41598_2022_21412_MOESM1_ESM.docx]

,;

**Supplementary Material for**

The dominant mesopredator and savanna formations shape the distribution of the rare northern tiger cat (*Leopardus tigrinus*) in the Amazon

Tadeu G. de Oliveira^1,2,3^*, Lester A. Fox-Rosales^3,4^, Evi A. D. Paemelaere^5,6^, Katia Maria Paschoaletto Micchi de Barros Ferraz^7^

^1^ Departamento de Biologia, Universidade Estadual do Maranhão (UEMA), Campus Paulo VI, Av. Lourenço Vieira da Silva 1000, Jardim São Cristóvão, São Luís, Maranhão, 65055-310, Brazil

^2^ Instituto Pró-Carnívoros, Av. Horácio Neto, 1020, Parque Edmundo Zanoni, Atibaia, São Paulo, 12945-010, Brazil

^3^ Programa de Pós-Graduação em Ecologia e Conservação da Biodiversidade PPGECB & PPG Ciência Animal da Universidade Estadual do Maranhão, Cidade Universitária Paulo VI, Av. Lourenço Vieira da Silva, nº 1000 – Bairro: Jardim São Cristóvão, CEP: 65055-310 – São Luís – MA, Brazil

^4^ Department of Conservation Biology, Georg-August Universität, Bürgerstrasse 50, 37073, Göttingen, Germany

^5^ Panthera, 8 W 40^th^ St. 18^th^ floor, New York, NY, 10018, USA

^6^ People & Wildlife Solutions, Manari, Region 9, Guyana

^7^ Departamento de Ciências Florestais, Escola Superior de Agricultura Luiz de Queiroz, Universidade de São Paulo, Avenida Pádua Dias 11, Piracicaba, São Paulo, 13418-900, Brazil

*Tadeu Gomes de Oliveira

Email: [tadeu4@yahoo.com](mailto:tadeu4@yahoo.com)

**Camera trapping in the Rupununi**

The camera-trapping study site comprised an area of seasonally flooded savanna ^1^, located on 300 km^2^ of private property belonging to the Karanambu Ranch of Rupununi, Guyana. Land use included ecotourism and noncommercial ranching, with approximately 100 head of cattle, as well as some subsistence hunting.

Cameras were placed at 41 stations (1 camera per station) spaced 1.5 km apart in five grid blocks, with a total polygon size of 145.4 km^2^, and recorded from February to August 2011, covering both the dry and wet seasons. To calculate the effective sampling area, we added a buffer of 13.06 km, which is the average mean maximum distance moved (MMDM) by small cats in areas where the ocelot is the dominant species ^2^.

The trapping effort was 1,974 trap-days, and the N-tiger cat RAI was 0.05 ind/100 trap-days. Converting this RAI into a density estimate based on the regression equation of the RAI to density of small felids and their proportions (de Oliveira et al., 2021A) yields a density estimate of 0.0014 ind/km^2^. This expected density of N-tiger cats for the Rupununi savanna was very low. As the mean density of small Neotropical cats is 4.52% of their RAI (n = 19) ^2^, the expected Rupununi density would be 0.002 ind/km^2^. Both density estimates are very low and are equivalent (0.0014 and 0.002 N-tiger cat/km^2^), representing the only estimates so far for the species in the Amazon biome. Moreover, these values were also similar to those found for other small cats in areas where ocelots are abundant ^2–4^. The relative abundance of N-tiger cats estimated in the rainforest at the Central Suriname Nature Reserve, an RAI of 0.02 ind/100 trap-days ^5^, was not confirmed to correspond to N-tiger cats after inspection of the original records ^a^. We estimated the ocelot RAI at 0.01 ind/100 trap-days and estimated a crude density in the Rupununi savanna of approximately 0.01–0.02 ind/km^2^, based on the density predicted by the model, which was an order of magnitude higher than the predicted density of N-tiger cats. Thus, the ocelot:tiger cat RAI ratio was 10:1 at that location. Although the information available is still scarce and very limited, it does provide a good glimpse into the reality expected for the tiger cat in the region and, to date, is the only information confirmed to be related to tiger cat camera-trap abundance estimates in Amazonia.

^a^ A.L.S. Gonçalves, pers. comm., T.G.de Oliveira, pers. obs.


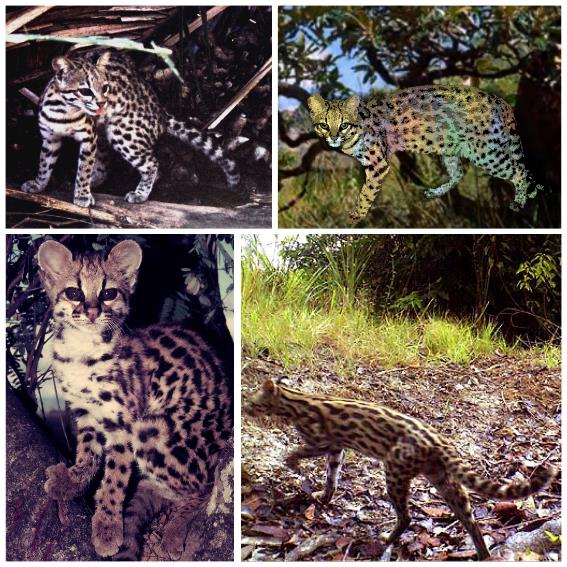


Fig. S1. Coloration patterns of N-tiger cat (*Leopardus tigrinus tigrinus*) specimens observed in the following Amazon rainforest and savanna locations: (A) deforestation arch in the eastern Amazonian transitional zone with babaçu forest (Brazil), (B) Carajás canga (an edaphic savanna; Brazil), (C) Gran Sabana (Venezuela), and (D) the Rupununi savanna (Guiana).

**
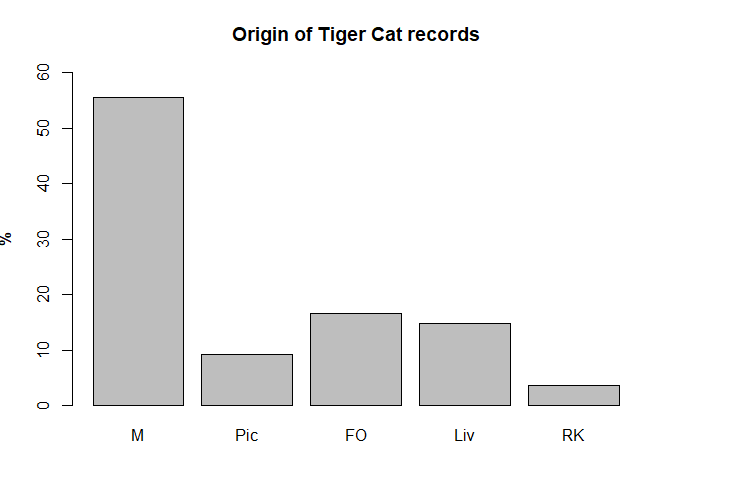
**

Fig. S2. Northern tiger cat records by origin. M = museum; Pic = pictures of live animals; FO = Field observations, visualizations, and tracks; Liv = Live animals trapped and released or apprehended from the exotic animal trade; RK = Roadkill.


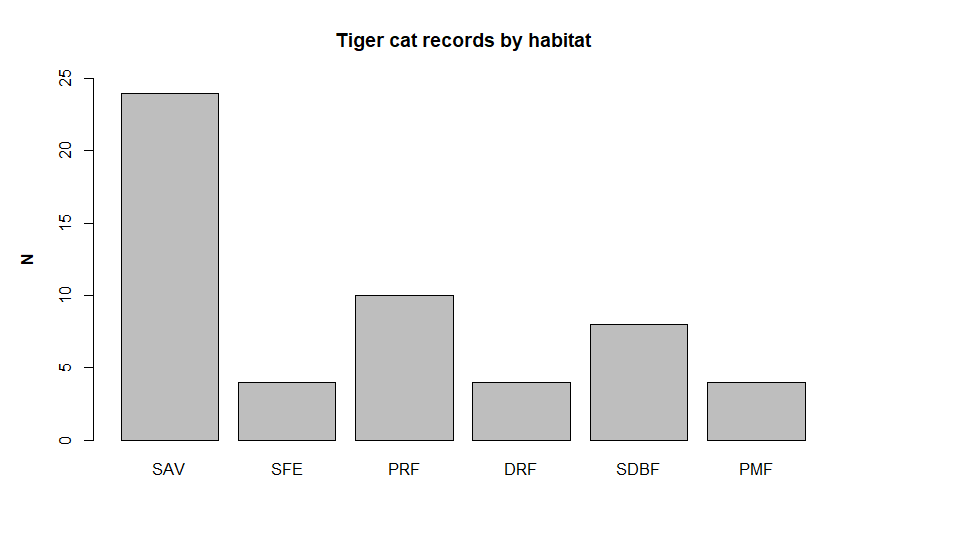


Fig. S3. Northern tiger cat records by habitat type. SAV = savanna; SFE = savana-forest ecotone; PRF = pristine rainforest; DRF = disturbed rainforest; SDBF = secondary deciduous forest and babaçú palm forest; PMF = pre-montane forest.


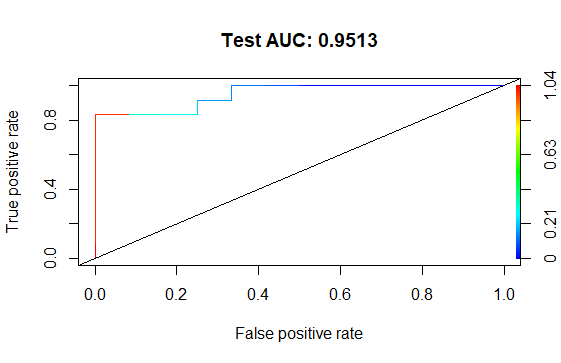


Fig. S4. Area under the receiver operating characteristic curve (AUC) for the best logistic regression model of Northern tiger cat presence.


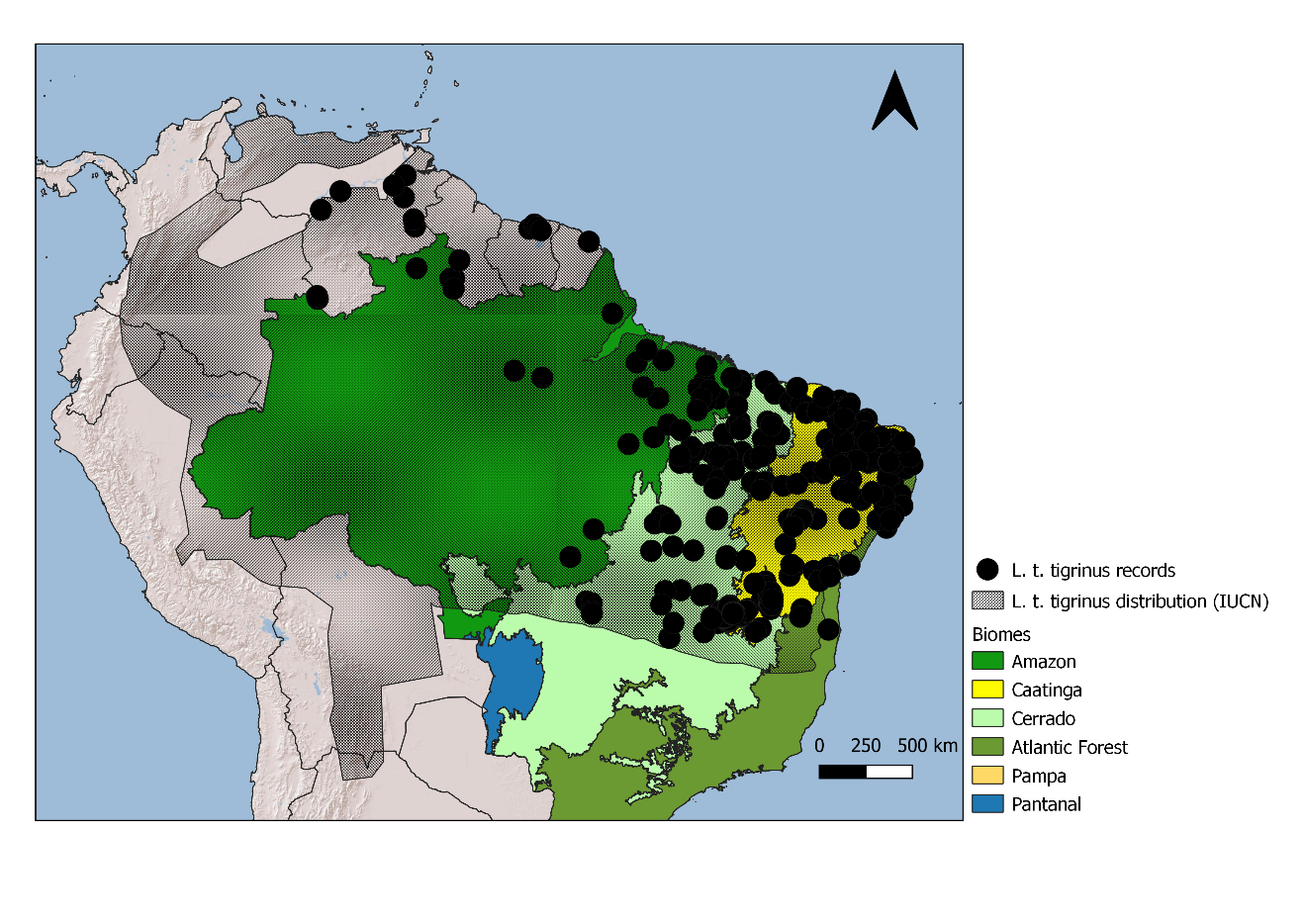


Fig. S5. Records of *L. tigrinus tigrinus* with the Brazilian biomes and the species distribution polygon based on the IUCN. Most records come from the Caatinga and Cerrado biomes. Figure made on QGIS ver. 3.4 ([www.qgis.org](http://www.qgis.org)).


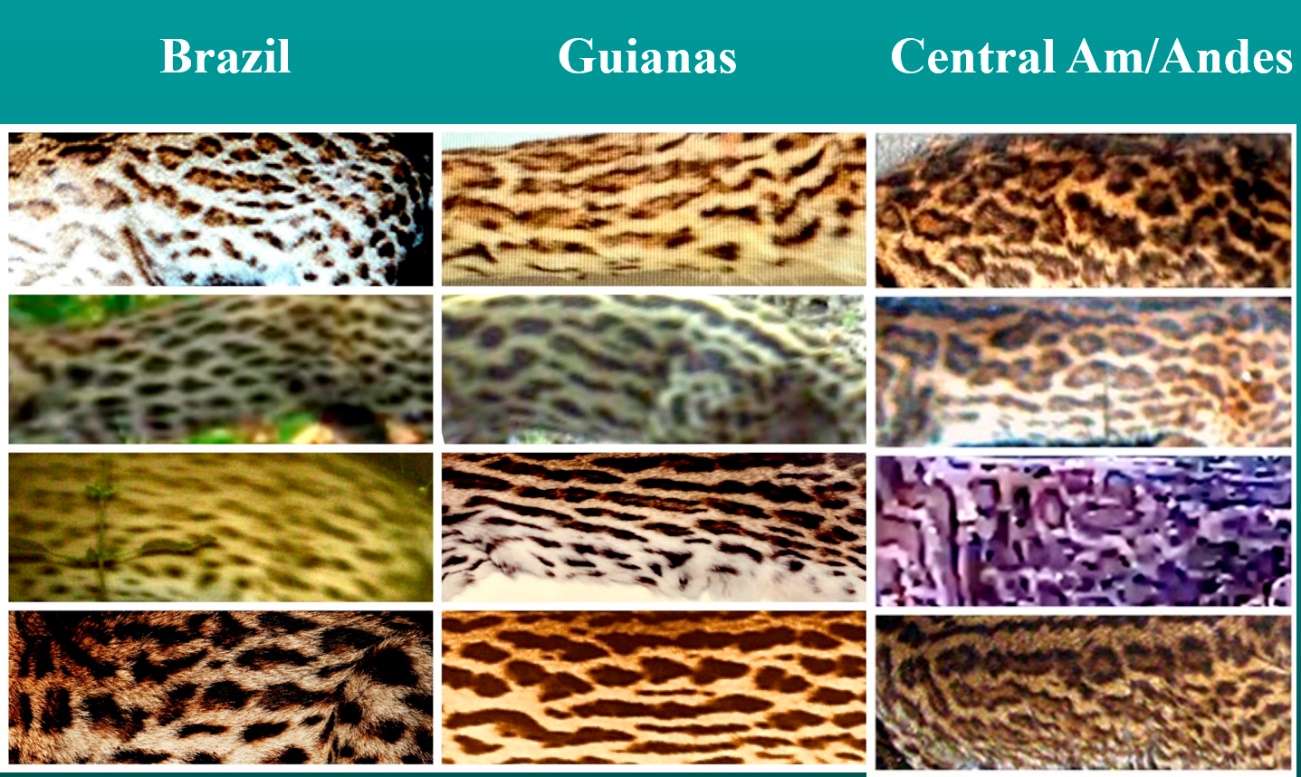


Fig. S6. Skin patterns of Northern tiger cat specimens from the Guianas compared to those from Brazil, Central America and the tropical Andes.


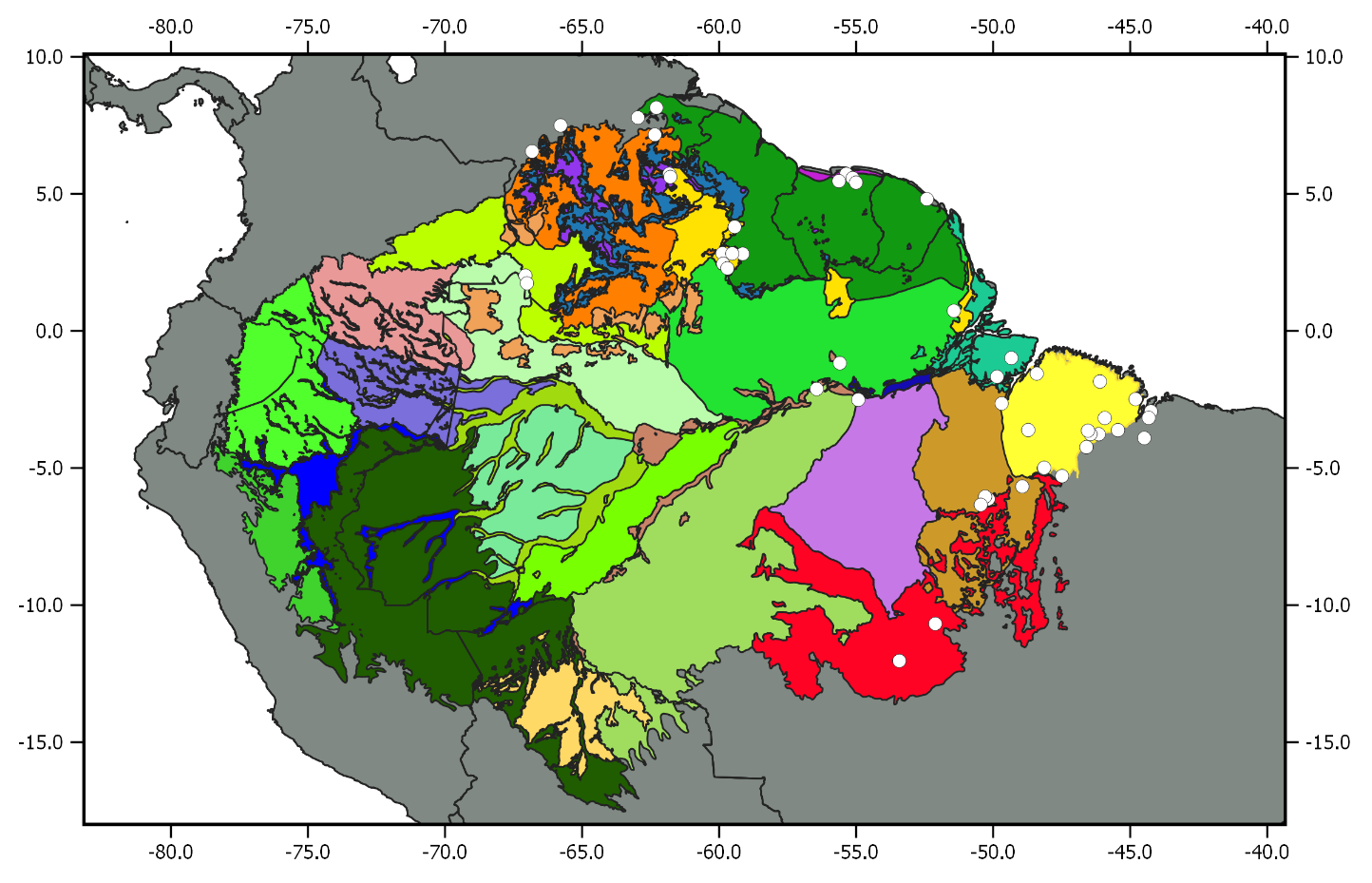


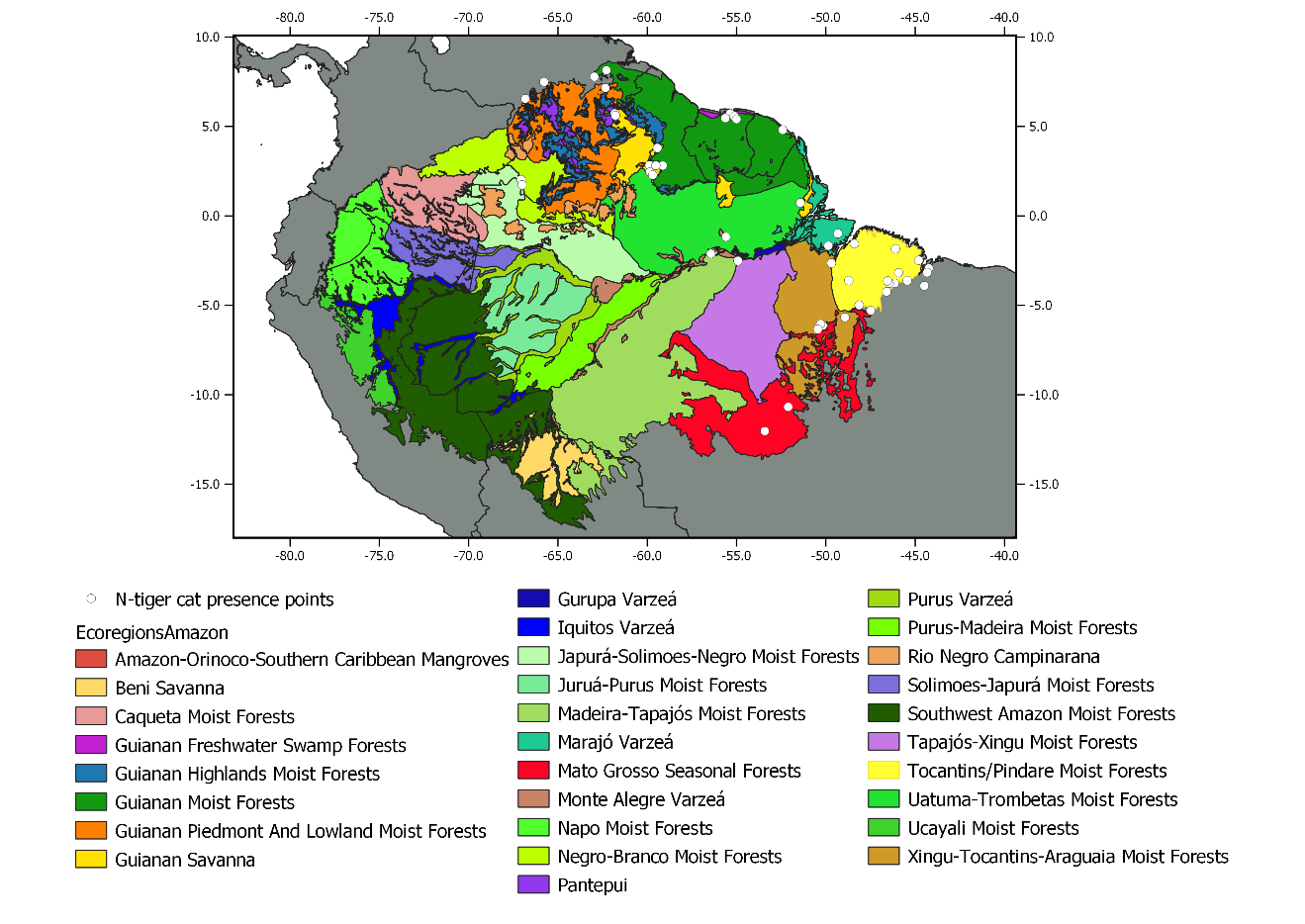


Fig. S7. Northern tiger cat presence in different Amazon ecoregions. Figure made on QGIS ver. 3.4 ([www.qgis.org](http://www.qgis.org)).

Table S1. Number (N) of Northern tiger cat records per ecoregion.

| **Ecoregion** | **N** |
| --- | --- |
| Tocantins-Pindaré moist forests | 12 |
| Guianan savannas | 10 |
| Guianan moist forests | 6 |
| Xingú-Tocantins Araguaia moist forests | 5 |
| Guianan Highlands moist forests | 3 |
| Maranhão Babaçú forests | 3 |
| Negro-Branco moist forests | 2 |
| Mato Grosso seasonal forest | 2 |
| Marajó várzea | 2 |
| Cerrado | 1 |
| Uatumá-Trombetas moist forests | 1 |
| Monte-Alegre várzea | 1 |
| Tapajós-Xingú moist forests | 1 |

Table S2. Survey effort (in trap-nights) and location of absence points used for modeling Northern tiger cat distribution in the Amazon biome.

| **Ecoregion** | **Number of absence points** | **Total survey effort (trap-nights)** | **Source** |
| --- | --- | --- | --- |
| Uatuma-Trombetas moist forests | 8 | 54,930 | ^6–10^ |
| Southwest Amazon moist forests | 7 | 31,976 | ^5,8,10–13^ |
| Napo moist forests | 6 | 39,542 | ^5,14,15^ |
| Solimoes-Japurá moist forests | 1 | 1,937 | ^16^ |
| Juruá-Purús moist forests | 2 | 16,049 | ^17–19^ |
| Purús-Madeira moist forests | 2 | 12,179 | ^9,10^ |
| Madeira-Tapajós moist forests | 5 | 32,607 | ^8,10^ |
| Mato Grosso seasonal forests | 3 | 12,760 | ^8,20^ |
| Tapajós-Xingu moist forests | 1 | 7,460 | ^10^ |
| Xingu-Tocantins-Araguaia moist forests | 2 | 14,975 | ^5,21^ |
| Purús várzea | 1 | 2,040 | ^22^ |
| Guianan moist forests | 5 | 47,830 | ^10,23^ ^1^ |
| Guianan freshwater swamp forests | 1 | 2,449 | ^23^ |
| Japurá-Solimões-Negro moist forests | 2 | 12,204 | ^10,22^ |
| Tocantins-Pindaré moist forests | 3 | 23,410 | ^10^ |
| **Total** | 49 | 312,348 | - |

1 Sanderson J., unpublished data.

**Table S3.** Ocelot densities used in the analysis.

| **Study Site** | **Ocelot Density**  **(ind/km^2^)** | **Reference** |
| --- | --- | --- |
| Block 39, Perú | 0.85 | ^15^ |
| Reserva Amanã | 0.29 | ^24^ |
| Tiputini Research Station | 0.63 | ^25^ |
| Morro do Diabo SP | 0.31 | ^26^ |
| UNIDERP | 0.56 | ^27^ |
| Estância Ecológica SESC Pantanal | 0.112 | ^27^ |
| Ravelo, Kaa-lya del Gran Chaco National Park | 0.59 | ^28^ |
| San Miguelito, Kaa-lya del Gran Chaco National Park | 0.56 | ^28^ |
| Cerro Cortado, Kaa-lya del Gran Chaco National Park | 0.30 | ^28^ |
| Tucavaca, Kaa-lya del Gran Chaco National Park | 0.29 | ^28^ |
| Yturria Ranch | 0.3 | ^29^ |
| Urugua-í | 0.13 | ^30^ |
| Iguazú National Park | 0.20 | ^30^ |
| Chiquibul Forest Reserve and National Park | 0.26 | ^31^ |
| Mountain Pine Ridge Forest Reserve | 0.03 | ^31^ |
| Ilha do Cardoso State Park | 0.4 | ^32^ |
| Feliciano Miguel Abdala Reseve | 0.29 | ^33^ |
| Sete Saloes | 0.06 | ^33^ |
| Iguaçú/ Iguazú National Parks and San Jorge Forest Reserve | 0.17 | ^34^ |
| Yaboti Biosphere Reserve | 0.09 | ^34^ |
| Caraguatá Reserve | 0.04 | ^35^ |
| Ponte Branca- ESEC MPL | 0.17 | ^36^ |
| Seis R | 0.25 | ^36^ |
| Santa Mônica | 0.62 | ^36^ |
| Darién National Park | 0.63 | ^37^ |
| Talamanca Caribbean Biological Corridor | 0.065 | ^38^ |
| Sociedad Civil Palmarito Natural Reserve | 0.11 | ^39^ |
| Serra da Capivara National Park | 0.045 | ^40^ |
| Palmar, Kaa-lya del Gran Chaco National Park | 0.77 | ^41^ |
| Estación, Kaa-lya del Gran Chaco National Park | 0.11 | ^41^ |
| Cocha Cashu | 0.8 | ^42^ |
| Turvo State Park (Porto) | 0.66 | ^43^ |
| Turvo State Park (Salto) | 0.38 | ^43^ |
| Laguna Atascosa | 0.09 | ^44^ |
| Mirador-Río Azul | 0.15 | ^45^ |
| Lorocachi | 0.93 | ^46^ |
| Maxus Road | 0.85 | ^46^ |
| Los Chimalapas | 0.38 | ^47^ |
| Hato Massaragual | 0.4 | ^48^ |
| Iguaçu NP | 0.14 | ^49^ |
| Corcovado NP | 0.24 | ^50^ |
| Chamela-Cuixmala | 0.39 | ^51^ |
| Medina, Colombia | 0.47 | ^52^ |
| Rio Bravo Conservation Area | 0.39 | ^53^ |
| Miranda Ranch | 0.76 | ^54^ |

Table S4. List of candidate variables used for spatial analyses.

| **Code** | **Variable** | **Resolution** | **Reference** | **Links** |
| --- | --- | --- | --- | --- |
| BIO1 | Annual Mean Temperature | 30 arcsec | ^55^ | 1 |
| BIO2 | Mean Diurnal Range | 30 arcsec | ^55^ | 1 |
| BIO3 | Isothermality | 30 arcsec | ^55^ | 1 |
| BIO4 | Temperature Seasonality | 30 arcsec | ^55^ | 1 |
| BIO5 | Max. Temperature of the Warmest Month | 30 arcsec | ^55^ | 1 |
| BIO6 | Min. Temperature of the Coldest Month | 30 arcsec | ^55^ | 1 |
| BIO7 | Annual Temperature Range | 30 arcsec | ^55^ | 1 |
| BIO8 | Mean Temperature of the Wettest Quarter | 30 arcsec | ^55^ | 1 |
| BIO9 | Mean Temperature of the Driest Quarter | 30 arcsec | ^55^ | 1 |
| BIO10 | Mean Temperature of the Warmest Quarter | 30 arcsec | ^55^ | 1 |
| BIO11 | Mean Temperature of the Coldest Quarter | 30 arcsec | ^55^ | 1 |
| BIO12 | Annual Precipitation | 30 arcsec | ^55^ | 1 |
| BIO13 | Precipitation of the Wettest Month | 30 arcsec | ^55^ | 1 |
| BIO14 | Precipitation of the Driest Month | 30 arcsec | ^55^ | 1 |
| BIO15 | Precipitation Seasonality | 30 arcsec | ^55^ | 1 |
| BIO16 | Precipitation of the Wettest Quarter | 30 arcsec | ^55^ | 1 |
| BIO17 | Precipitation of the Driest Quarter | 30 arcsec | ^55^ | 1 |
| BIO18 | Precipitation of the Warmest Quarter | 30 arcsec | ^55^ | 1 |
| BIO19 | Precipitation of the Coldest Quarter | 30 arcsec | ^55^ | 1 |
| TC | Percent Tree Cover | 30 arcsec | ^56^ | 2 |
| ELEV | Mean Altitude above Sea Level | 30 arcsec | ^57^ | 3 |
| NPP | Mean Net Primary Productivity | 30 arcsec | ^58^ | 4 |
| HEIGHT | Mean Canopy Height | 30 arcsec | ^59^ | 5 |
| SAV | Categorical Habitat  (1 = record is from the savanna; 0 = not from the savanna) | 30 arcsec | ^60^ | 6 |
| LPDens | Mean Potential Ocelot Density | 30 arcsec | This work |  |

**1** <https://www.worldclim.org/>

**2** <https://earthenginepartners.appspot.com/science-2013-global-forest>

**3** <https://earthexplorer.usgs.gov/>

**4** <http://www.ntsg.umt.edu/project/modis/mod17.php>

**5** <https://glad.umd.edu/dataset/gedi>

**6** <https://amazonia.mapbiomas.org/en>

**SI References**

1. Cummings, A. R., Read, J. M. & Fragoso, J. M. V. Utilizing Amerindian Hunters’ Descriptions to Guide the Production of a Vegetation Map. *Int. J. Appl. Geospatial Res.* **6**, 118–142 (2015).

2. de Oliveira, T. G. *et al.* *Assessing Small Cats Abundance in Brazil: Camera Trapping Summary Report - 2018*. https://procarnivoros.org.br/projeto/gatos-do-mato-brasil/ (2019).

3. de Oliveira, T. G. *et al.* A refined population and conservation assessment of the elusive and endangered northern tiger cat (Leopardus tigrinus) in its key worldwide conservation area in Brazil. *Glob. Ecol. Conserv.* **22**, (2020).

4. de Oliveira, T. G. *et al.* Demographic and spatial patterns of small felids in Brazil and the ocelot effect. in *100th Annual Meeting of the American Society of Mammalogists* (Even3Publicações, 2021). doi:http://doi.org/10.29327/765836.

5. Santos, F. *et al.* Prey availability and temporal partitioning modulate felid coexistence in Neotropical forests. *PLoS One* **14**, 1–23 (2019).

6. Benchimol, M. & Peres, C. A. Widespread forest vertebrate extinctions induced by a mega hydroelectric dam in lowland Amazonia. *PLoS One* (2015) doi:10.1371/journal.pone.0129818.

7. De Luna, R. B., Alfonso Reyes, A. F., De Lucena, L. R. R. & Pontes, A. R. M. Terrestrial mammal assemblages in protected and human impacted areas in Northern Brazilian Amazonia. *Nat. Conserv.* (2017) doi:10.3897/natureconservation.22.17370.

8. de Oliveira, T. G. *et al.* How rare is rare? Quantifying and assessing the rarity of the bush dog Speothos venaticus across the Amazon and other biomes. *ORYX* (2018) doi:10.1017/S0030605316000624.

9. Wang, B. *et al.* Habitat use of the ocelot (Leopardus pardalis) in Brazilian Amazon. *Ecol. Evol.* **9**, 5049–5062 (2019).

10. Gonçalves, A. L. S. Padrões espaço-temporais na ocorrência de pequenos felinos amazônicos em resposta a fatores bióticos a abióticos. (Instituto Nacional de Pesquisas da Amazônia, 2022).

11. Gómez, H., Wallace, R. B., Ayala, G. & Tejada, R. Dry season activity periods of some Amazonian mammals. *Stud. Neotrop. Fauna Environ.* (2005) doi:10.1080/01650520500129638.

12. Tobler, M. W., Carrillo-Percastegui, S. E., Leite Pitman, R., Mares, R. & Powell, G. An evaluation of camera traps for inventorying large- and medium-sized terrestrial rainforest mammals. *Anim. Conserv.* (2008) doi:10.1111/j.1469-1795.2008.00169.x.

13. Borges, L. H. M., Calouro, A. M. & de Sousa, J. R. D. Large and medium-sized mammals from chandless State Park, Acre, Brazil. *Mastozool. Neotrop.* (2015).

14. Espinosa, S., Celis, G. & Branch, L. C. When roads appear jaguars decline: Increased access to an Amazonian wilderness area reduces potential for jaguar conservation. *PLoS One* (2018) doi:10.1371/journal.pone.0189740.

15. Kolowski, J. M. & Alonso, A. Density and activity patterns of ocelots (Leopardus pardalis) in northern Peru and the impact of oil exploration activities. *Biol. Conserv.* (2010) doi:10.1016/j.biocon.2009.12.039.

16. Payán, E. Hunting sustainability, species richness and carnivore conservation in Colombian Amazonia. *Dep. Biol. Anthropol.* (2009).

17. Pimenta, C. S. Uso de habitat e ocupação por carnívoros em uma Reserva de Uso Sustentável na Amazônia Central, Brasil. (Instituto Nacional da Pesquisas da Amazônia, 2012).

18. Botelho, A. L. M., Calouro, A. M., Borges, L. H. M. & Chaves, W. A. Large and medium-sized mammals of the Humaitá Forest Reserve, southwestern Amazonia, state of Acre, Brazil. *Check List* (2012) doi:10.15560/8.6.1190.

19. Costa, H. C. M., Peres, C. A. & Abrahams, M. I. Seasonal dynamics of terrestrial vertebrate abundance between Amazonian flooded and unflooded forests. *PeerJ* **6**, e5058 (2018).

20. Negrões, N. *et al.* Private forest reserves can aid in preserving the community of medium and large-sized vertebrates in the Amazon arc of deforestation. *Biodivers. Conserv.* (2011) doi:10.1007/s10531-010-9961-3.

21. Carvalho, A. S. *et al.* Large and medium-sized mammals of Carajás National Forest, Pará State, Brazil. *Check List* (2014) doi:10.15560/10.1.1.

22. Alvarenga, G. C. *et al.* Spatial patterns of medium and large size mammal assemblages in várzea and terra firme forests, Central Amazonia, Brazil. *PLoS One* **13**, 1–19 (2018).

23. Schuttler, S. *et al.* Can mammals thrive near urban areas in the Neotropics? Characterizing the community of a reclaimed tropical forest. *Trop. Ecol.* **62**, 174–185 (2021).

24. Rocha, D. G. da, Sollmann, R., Ramalho, E. E., Ilha, R. & Tan, C. K. W. Ocelot (Leopardus pardalis) Density in Central Amazonia. *PLoS One* **11**, e0154624 (2016).

25. Mosquera, D., Blake, J. G., Swing, K. & Romo, D. Ocelot ( Leopardus pardalis ) density in Eastern Ecuador based on capture–recapture analyses of camera trap data. *Neotrop. Biodivers.* **2**, 51–58 (2016).

26. Jacob, A. A. Ecologia e conservação da jaguatirica (Leopardus pardalis) no Parque Estadual Morro do Diabo, Pontal do Paranapanema, SP. (Universidade de Brasilia, 2002).

27. Trolle, M. & Kéry, M. ESTIMATION OF OCELOT DENSITY IN THE PANTANAL USING CAPTURE–RECAPTURE ANALYSIS OF CAMERA-TRAPPING DATA. *J. Mammal.* **84**, 607–614 (2003).

28. Maffei, L., Noss, A. J., Cuéllar, E. & Rumiz, D. I. Ocelot ( Felis pardalis ) population densities, activity, and ranging behaviour in the dry forests of eastern Bolivia: data from camera trapping. *J. Trop. Ecol.* **21**, 349–353 (2005).

29. Haines, A. M., Janecka, J. E., Tewes, M. E., Grassman Jr, L. I. & Morton, P. The importance of private lands for ocelot Leopardus pardalis conservation in the United States. *Oryx* **40**, 90–94 (2006).

30. Di Bitetti, M. S., Paviolo, A. & De Angelo, C. Density, habitat use and activity patterns of ocelots (Leopardus pardalis) in the Atlantic Forest of Misiones, Argentina. *J. Zool.* (2006) doi:10.1111/j.1469-7998.2006.00102.x.

31. Dillon, A. & Kelly, M. J. Ocelot Leopardus pardalis in Belize: the impact of trap spacing and distance moved on density estimates. *Oryx* **41**, 469–477 (2007).

32. Costa, R. F. Levantamento populacional da jaguatirica (Leopardus pardalis), através do uso de armadilhas fotográficas no Parque Estadual Ilha do Cardoso, litoral sul do Estado de São Paulo. (Universidade de São Paulo, 2007).

33. Massara, R. L., Paschoal, A. M. de O., Doherty, P. F., Hirsch, A. & Chiarello, A. G. Ocelot Population Status in Protected Brazilian Atlantic Forest. *PLoS One* **10**, e0141333 (2015).

34. Di Bitetti, M. S., Paviolo, A., De Angelo, C. D. & Di Blanco, Y. E. Local and continental correlates of the abundance of a neotropical cat, the ocelot (Leopardus pardalis). *J. Trop. Ecol.* **24**, 189–200 (2008).

35. Goulart, F. V. B. *et al.* Habitat selection by large mammals in a southern Brazilian Atlantic Forest. *Mamm. Biol.* (2009) doi:10.1016/j.mambio.2009.02.006.

36. Lima, F. ESTIMATIVAS DE ABUNDÂNCIA E DENSIDADE POPULACIONAL DA JAGUATIRICA ATRAVÉS DE MODELOS DE MARCAÇÃO-RECAPTURA: ESTUDO DE CASO NOS REMANESCENTES FLORESTAIS DO PONTAL DO PARANAPANEMA, SÃO PAULO. (Pontifícia Universidade Católica de Minas Gerais, 2009).

37. Moreno, R. & Bustamante, A. DATOS ECOLOGICOS DEL OCELOTE (Leopardus pardalis) EN CANA, PARQUE NACIONAL DARIEN, PANAMA; UTILIZANDO EL METODO DE CAMARAS TRAMPA. *Tecnociencia* **11**, 91–102 (2009).

38. González-Maya, J. F. & Cardenal-Porras, J. Ocelot density in the Caribbean slope of the Talamanca region, Costa Rica. *Hystrix* **22**, 355–360 (2011).

39. Díaz-Pulido, A. & Payán, E. Densidad de ocelotes (Leopardus pardalis) en los llanos colombianos. *Mastozoología Neotrop.* **18**, (2011).

40. Oliveira, G. P. de. Ecologia da Jaguatirica, Leopardus Pardalis (linnaeus, 1758), na caatinga do Piauí. (Universidade de Brasília, 2012).

41. Noss, A. J. *et al.* Comparison of density estimation methods for mammal populations with camera traps in the Kaa-Iya del Gran Chaco landscape. *Anim. Conserv.* **15**, 527–535 (2012).

42. Emmons, L. H. A field study of ocelots (Felis pardalis) in Peru. *Rev. d’écologie* (1988).

43. Kasper, C. B., Mazim, F. D., Soares, J. B. G. & Oliveira, T. G. de. Density estimates and conservation of Leopardus pardalis southernmost population of the Atlantic Forest. *Iheringia. Série Zool.* **105**, 367–371 (2015).

44. Sternberg, M. A. & Mays, J. L. *Ocelot Survey in and around Laguna Atascosa National Wildlife Refuge*. (2011).

45. Moreira, J. *et al.* *Densidad de ocelotes (Carnívora Leopardus pardalis) en la parte este del Parque Nacional Mirador Río Azul, Guatemala*. (2007).

46. Salvador, J. & Espinosa, S. Density and activity patterns of ocelot populations in Yasuní National Park, Ecuador. *Mammalia* **80**, (2016).

47. Zeller, K. A., Nijhawan, S., Salom-Pérez, R., Potosme, S. H. & Hines, J. E. Integrating occupancy modeling and interview data for corridor identification: A case study for jaguars in Nicaragua. *Biol. Conserv.* (2011) doi:10.1016/j.biocon.2010.12.003.

48. Ludlow, M. E. & Sunquist, M. E. *Ecology and behavior of ocelots in Venezuela*. (1987).

49. Crashaw, P. G. Comparative ecology of ocelot (Felis pardalis) and jaguar (Panthera onca) in a protected subtropical forest in Brazil and Argentina. (University of Florida, 1995).

50. Salom-Pérez, R. Ecología del jaguar (Panthera onca) y del manigordo (Leopardus pardalis) (Carnivora: Felidae) en el Parque Nacional Corcovado, Costa Rica. (Universidad de Costa Rica, 2005).

51. Fernández, E. C. Ocelot (Leopardus Pardalis) Ecology in the Chamela-Cuixmala Biosphere Reserve, Jalisco, Mexico. (University of Wyoming, 2002).

52. Valderrama-Vasquez, C. Densidad de ocelote y abundancias relativas de ocelote y margay, usando datos de cámaras trampa en la cordillera oriental de los Andes colombianos. in *Grandes Felinos de Colombia* (eds. Payán, E. & Castano-Uribe, C.) 132–144 (Panthera, Conservación Internacional, Fundación Herencia Ambiental Caribe, 2013).

53. Davis, M. L. Densities, habitat-use, and mesopredator release of the ocelot in Belize. (Virginia Polytechnic Institute and State University, 2008).

54. Crawshaw, P. G. & Quigley, H. B. Notes on Ocelot Movement and Activity in the Pantanal Region, Brazil. *Biotropica* **21**, 377 (1989).

55. Fick, S. E. & Hijmans, R. J. WorldClim 2: new 1‐km spatial resolution climate surfaces for global land areas. *Int. J. Climatol.* **37**, 4302–4315 (2017).

56. Hansen, M. C. *et al.* High-Resolution Global Maps of 21st-Century Forest Cover Change. *Science (80-. ).* **342**, 850–853 (2013).

57. USGS. USGS EROS Archive - Digital Elevation - Shuttle Radar Topography Mission (SRTM) 1 Arc-Second Global. https://www.usgs.gov/centers/eros/science/usgs-eros-archive-digital-elevation-shuttle-radar-topography-mission-srtm-1-arc?qt-science_center_objects=0#qt-science_center_objects (2018).

58. Zhao, M., Heinsch, F. A., Nemani, R. R. & Running, S. W. Improvements of the MODIS terrestrial gross and net primary production global data set. *Remote Sens. Environ.* **95**, 164–176 (2005).

59. Potapov, P. *et al.* Mapping global forest canopy height through integration of GEDI and Landsat data. *Remote Sens. Environ.* **253**, 112165 (2021).

60. Souza, C. M. *et al.* Reconstructing Three Decades of Land Use and Land Cover Changes in Brazilian Biomes with Landsat Archive and Earth Engine. *Remote Sens.* **12**, 2735 (2020).
